# Supplementary material for: System drift in the evolution of plant meristem development
Source: PLoS Genet. 2026 Apr 3;22(4):e1012089. doi: 10.1371/journal.pgen.1012089 (PMC13075796; doi:10.1371/journal.pgen.1012089)
Supplement: S1 Appendix — (PDF) [file pgen.1012089.s015.pdf]

Appendix S1 Drift vs adaptation

To observe DSD, we cloned our populations at the apparent fitness plateau at 50,000 generations. In the subsequent 50,000 generations, we observed a very slight fitness increase in most populations (Fig S3). To determine if this increase was due to specific adaptive mutations, we analyzed the probability that a mutation with a given fitness increase a would become fixed within the population versus the probability that a mutation providing no fitness gain would become fixed. For a mutation to become fixed the given lineage has to be selected for offspring in successive generations.

The probability of an individual to be picked for generating offspring is $\frac{f_q}{\sum_i^N f_i}$ for a single trial. An individual with a mutation resulting in fitness gain a will have probability $p_q = \frac{f_q + a}{(\sum_i^N f_i) + a}$ of being picked, again for a single trial. For $a << f_i$ we can assume

$$\sum_i^N (f_i) + a \approx \sum_i^N (f_i + a) \approx \sum_i^N f_i, \quad \textbf{(eq. S1)}$$

which means that we can treat p_q as a constant instead of having to account for fitness gain of the total population. Our selection (roulette wheel selection) can be described as a Bernoulli process which has the following probability mass function

$$f(k,n,p) = Pr(X = k) = \binom{n}{k} p^k (1-p)^{n-k}, \quad \textbf{(eq. S2)}$$

describing the probability of exactly k successes in n trials with probability p success. For ease of notation we describe the probability of m individuals with the mutation to be present in generation g as $P_{m,g}$. Assuming only a single individual gains the mutation we can denote the probability vector of generation 0 as $P_{1,0} = 1$ and $P_{i,0} = 0 \forall i \neq 1$.

$$P_{m,g}(p_q) = \sum_i [P_{i,g-1} \cdot f(m,n,i \cdot p_q)]. \quad \textbf{(eq. S3)}$$

This function can also be adapted to allow for fitness gain over time through

$$p_q(g) = \frac{f_q + ag}{(\sum_i^N f_i)} \quad \textbf{(eq. S4)}$$

$$P_{m,g}(p_q(g)) = \sum_i [P_{i,g-1} \cdot f(m,n,i \cdot p_q(g))] \quad \textbf{(eq. S5)}$$

Now we can describe the probability that a given mutation is still present in the population at generation g as $1 - P_{0,g}$. This system converges to $P_g \approx P_{g-1}$. We investigated P_g after 1000 generations, or when $\mathrm{sum}(P_g - P_{g-1})^2 \leq 0.0001$, after which we call $1 - P_{0,1000}$ the fixation probability. If for a mutation with a given fitness increase a the fixation probability is significantly higher than for a mutation without that fitness increase, we assume a selective regime. If a fitness gain a results in a negligible increase in fixation probability we assume a neutral regime.

Using the data from simulation 2 we estimated the fitness gain at each time point by fitting a line $(ax + b = y)$ through the next 5 fitness values. As we only sampled the population every 100 generations this slope might be an underestimate of the actual fitness gain achieved. To get a best-case upper bound we therefore multiplied this slope by 100, which assumes that all the fitness gain within that window was gained in just 5 generations. At each generation we compute the log fold change of fixation probability

$$\log_2 \left[(1 - P_{0,g} \left(\frac{f_q + a}{(\sum_i^N f_i)} \right)) / (1 - P_{0,g} \left(\frac{f_q}{(\sum_i^N f_i)} \right)) \right], \quad \textbf{(eq. S6)}$$

for the given slope and for $a = 0$ as neutral baseline. Figure S15A shows the fitness of the population at intervals of 10,000 generations, and the median fitness of the population over time. Using the median population fitness we gain a at each timepoint and compute the fold change in fixation probability (Fig S15B). For example, in generation 12,500 the fixation probability of the genome of a median fit individual is 3.97$\cdot 10^{-7}$, compared to the fixation probability of the genome of a median fit individual plus fitness gain 0.36. Whereas the fixation probability of the genome of a median fit individual at generation 50,000 is 0.144 versus the probability with fitness gain 0.140 (a was slightly negative at this point). Only in the beginning of the simulation up until around generation 15,000 there is a notable difference in fixation probability. The fitness gain after generation 50,000 is too shallow for selection to outweigh drift. This method, however, only detects direct adaptive fitness gains and will miss out on higher order effects such as increases in mutational or developmental robustness. During the shallow fitness slope such higher order effects are much more likely to play a dominant role.

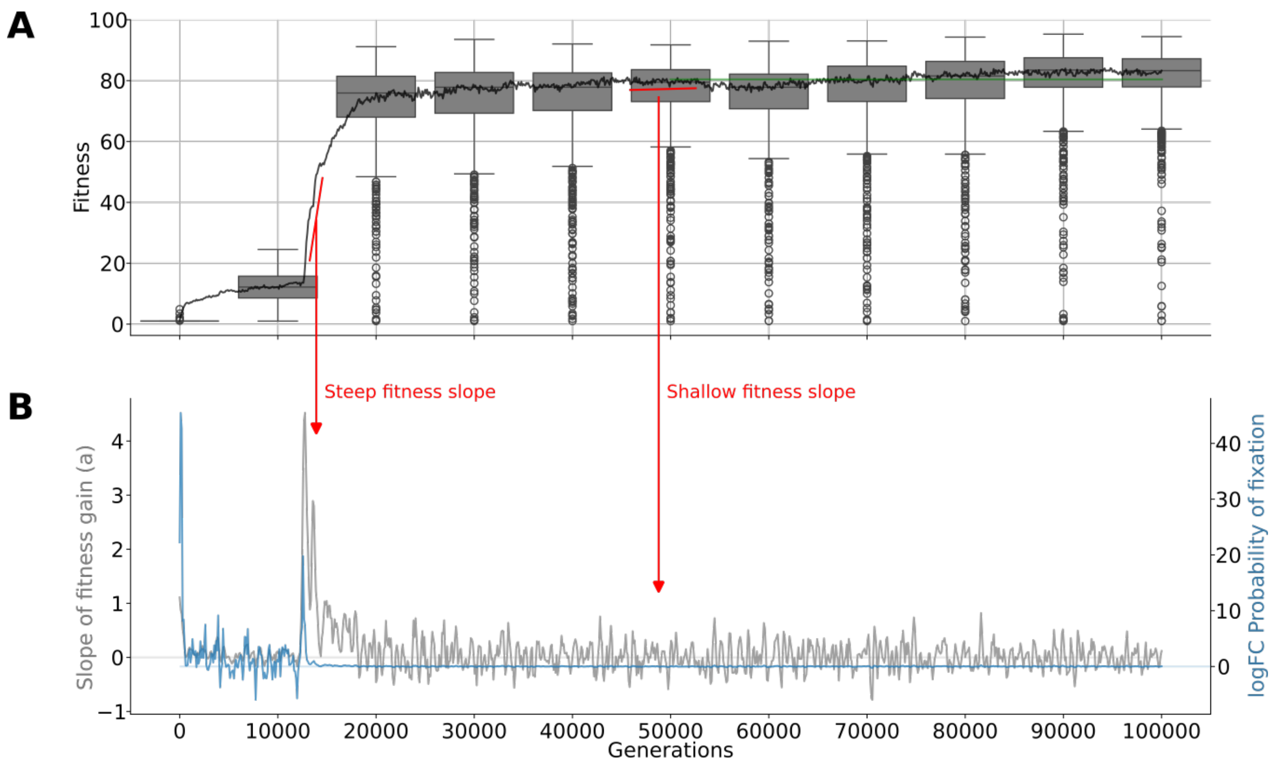

Figure S15. Neutral vs adaptive regimes of fitness gain. (A) Fitness of the whole population shown in box plots at intervals of 10,000 generations. Median fitness over generations shown per 100 generations as line. In green the median fitness of population at generation 50,000. **(B)** The slope of fitness gain per generation (grey) and the log2 fold change in fixation probability of a mutation providing fitness gain a versus without, see eq. S6.
