# Supplementary material for: System drift in the evolution of plant meristem development
Source: PLoS Genet. 2026 Apr 3;22(4):e1012089. doi: 10.1371/journal.pgen.1012089 (PMC13075796; doi:10.1371/journal.pgen.1012089)
Supplement: S2 Appendix — (PDF) [file pgen.1012089.s016.pdf]

Appendix S2 Evolution of Robustness during stabilising selection

We investigated the changes in either mutational robustness or developmental robustness over evolutionary time, especially during stabilising selection.

Mutational robustness (R_m) is defined as

$$R_m = \frac{\mathrm{med}(F_{\mathrm{offspring}}) - \mathrm{med}(F_{\mathrm{parent}})}{\mathrm{med}(F_{\mathrm{parent}})}, \qquad \textbf{(eq. S7)}$$

where $\mathrm{med}(F_{\mathrm{offspring}})$ denotes the median offspring fitness and $\mathrm{med}(F_{\mathrm{parent}})$ the median parent fitness. In other work, such as in [1], mutational robustness is described in terms of only the mutated offspring. However, the fluctuating genome sizes in our simulations result in different probabilities for offspring to be mutated. Only considering mutated offspring in Equation eq. S7 does not take into account this variability. On the other hand, considering all offspring might hide concomitant changes in developmental robustness.

Developmental robustness (R_d) is described as the variance proportional to the mean of clonal individuals. We transform this value such that a positive slope describes an increase in robustness.

$$R_d = \log\left[\frac{1}{1 + \frac{\mathrm{var}(F_{\mathrm{clones}})}{\mathrm{mean}(F_{\mathrm{clones}})}}\right] \qquad \textbf{(eq. S8)}$$

During stabilising selection (after generation 50,000), both the mutational and developmental robustness fluctuate, but neither does clearly increase or decrease overall (Fig S16). An exception to this is simulation 15_0 where a clearer gain in fitness can be observed at generation 90,000, which is also reflected in the distributions describing mutational and developmental robustness. This highlights the importance of neutral drift and how it interplays with adaptive steps, described before in [2]. In deterministic simulations divergence of the GRNs is still observed (Fig S4). Although this does not rule out a role for selection for developmental robustness in the divergence of the stochastic simulations, it does show that it is not a necessary driver of divergence. Reducing the size of the GRN made evolving developmental programs more challenging, showing the importance of available degrees of freedom in the genome (Fig S8). Nevertheless, similar neutral drift patterns can be seen in the mutational and developmental robustness.

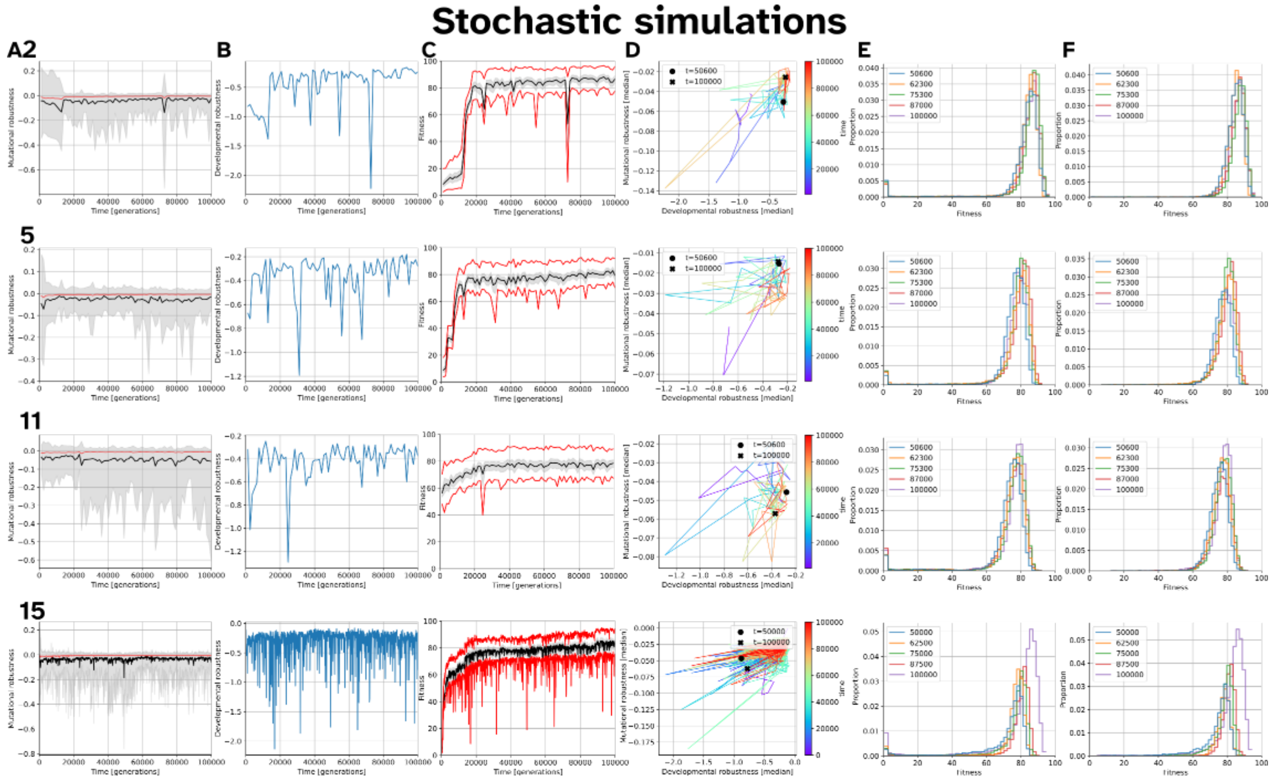

Figure S16. Stabilising selection of stochastic simulations. All plots show the stats of individuals along an ancestral lineage of stochastic simulations with their simulation ID indicated at the left of each row. **(A)** Mutational robustness (see eq. S7 over generational time. Red considers all offspring; black considers only the mutated offspring, IQR of the mutated offspring is shown in grey. **(B)** Developmental robustness (see eq. S8) over generational time. **(C)** Median fitness of clonal repeats (black) with IQR (grey) and upper 95% (red). **(D)** Mutational robustness plotted against developmental robustness for several timepoints along the line of descent (indicated by color). For mutational robustness we only considered mutated offspring. **(E)** Fitness distribution of 10,000 offspring. **(F)** Fitness distribution of only the non-mutated offspring in E.

References

1. Draghi JA, Parsons TL, Wagner GP, Plotkin JB. Mutational robustness can facilitate adaptation. Nature. 2010;463(7279):353-5.
2. Wagner A. Neutralism and selectionism: a network-based reconciliation. Nature Reviews Genetics. 2008;9(12):965-74.
