# Supplementary material for: System drift in the evolution of plant meristem development
Source: PLoS Genet. 2026 Apr 3;22(4):e1012089. doi: 10.1371/journal.pgen.1012089 (PMC13075796; doi:10.1371/journal.pgen.1012089)
Supplement: S3 Appendix — (PDF) [file pgen.1012089.s017.pdf]

Appendix S3 Divergence metrics

To measure the divergence of different GRNs we considered the following metrics: graph edit distance (GED), adjacency matrix difference, and Boolean adjacency matrix difference (see Fig S17). The GED is a measure of the minimal number of edits a graph needs to undergo in order to be similar to another graph. For the two matrix based comparisons we first transform the graph into two NxN matrices (one for activating and one for inhibiting regulatory interactions) for N unique gene types. If multiple copies of a gene exist, their incoming interactions of a specific type are summed. The difference between the adjacency matrix and the Boolean matrix is that any input > 0 in the adjacency matrix is set to 1 for the Boolean matrix. $\forall(i,j), M^B_{i,j} = \min(1, M^{adj}_{i,j})$ where M^{adj} denotes the adjacency matrix and M^B denotes the Boolean matrix.

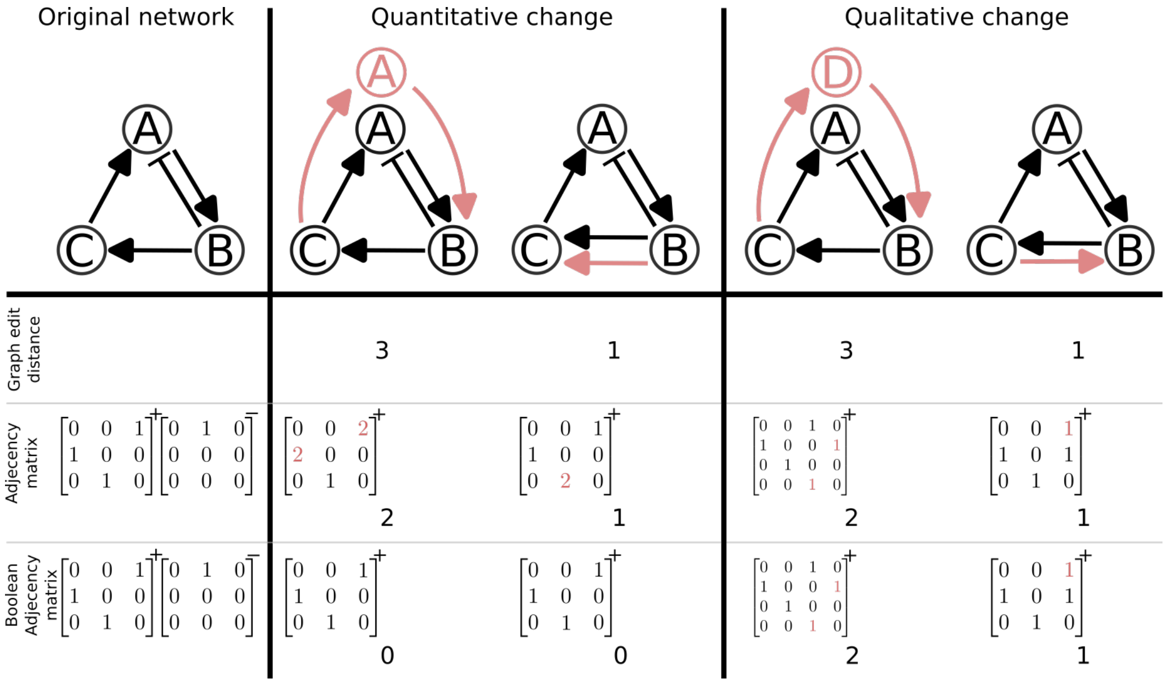

Figure S17. Different distance metrics for comparison of networks. The matrix notation of the networks also distinguishes activating $(+)$ and inhibiting $(-)$ interactions by storing them in separate matrices. The rows in the matrix represent genes and the columns by which gene they are activated/inhibited, so for example if gene 0 activates gene 2 then $M^+_{0,2} = 1$.
