# Supplementary material for: System drift in the evolution of plant meristem development
Source: PLoS Genet. 2026 Apr 3;22(4):e1012089. doi: 10.1371/journal.pgen.1012089 (PMC13075796; doi:10.1371/journal.pgen.1012089)
Supplement: S1 Table — (PDF) [file pgen.1012089.s018.pdf]

	Value	Unit	Use
**Mechanical parameters**			
x_bounds	[-61, 61]	[um, um]	Horizontal bounds of the tissue
y_bounds	[0, 40]	[um, um]	Vertical bounds of the system
F_bound	10	a.f.u.	Boundary force
F_spring	1	a.f.u.	Spring constant
eq_dist	0.9	um	Equillibrium distance of the spring
**Molecular parameters**			
decay mRNA (α)	0.2 [0.05, 0.9]	t^{-1}	Initial decay rate [min, max]
decay protein (β)	0.2	t^{-1}	Decay rate of proteins
connections	3		Initial avg nr of connections per gene
TFBS hill constant (H)	40 [5, 100]	a.m.u.	Initial Hill constant [min, max]
Max gene expression rate (τ)	25 [5, 80]	a.m.u. t^{-1}	Initial max expression rate [min, max]
Dimer k_{on}	0.01 [0.00001, 0.1]		Initial k_{on} of the dimer gene [min, max]
Dimer k_{off}	0.001 [0.00001, 0.1]		Initial k_{off} of the dimer gene [min, max]
Diffusion coefficient (D)	1 [0.5, 5]	um^2 t^{-1}	Diffusion coefficient [min, max]
**Simulation parameters**			
N_pop	1000		Population size
dev_steps	500		Developmental timesteps
generations	50000		Number of generations (initially)
N_genes	14		Number of unique genes
N_diff	2		Number of diffusing gene products
N_cellcell	2		Number of cellcell gene products
**Mutational parameters**			
gene duplication probability	0.002		per gene
gene deletion probability	0.003		per gene
gene parameter change	0.0005		Probability of changing decay mRNA or E
TFBS sign change	0.0002		per TFBS
TFBS type switch	0.0002		Probability of changing TFBS type per TFBS
TFBS duplication probability	0.0005		per TFBS
TFBS deletion probability	0.00075		per TFBS
TFBS innovation probability	0.00004 * genome size		max once per offspring
TFBS H change probability	0.002		per TFBS
Whole genome duplication probability	0.0005		per offspring
σ^2 max gene expression (τ) change	5		
σ^2 TFBS hill constant (H) change	10		
σ^2 mRNA decay rate (α) change	0.01		

Table S1. Table of the standard parameters used. a.f.u., arbitrary force unit. a.m.u., arbitrary molar unit. t, developmental time step.
