# Supplementary material for: System drift in the evolution of plant meristem development
Source: PLoS Genet. 2026 Apr 3;22(4):e1012089. doi: 10.1371/journal.pgen.1012089 (PMC13075796; doi:10.1371/journal.pgen.1012089)
Supplement: S2 Table — (PDF) [file pgen.1012089.s019.pdf]

Term	Abbreviation	Meaning
Developmental System Drift	DSD	The phenomenon where a trait maintains a constant phenotype but shows divergence in its underlying developmental pathway
Shoot Apical Meristem	SAM	Stem cell niche located in the apical tip of a (vascular) plant
Transcription Factor	TF	DNA binding protein which enhances/inhibits transcription
Transcription Factor Binding Site	TFBS	Site in the promoter where a TFBS can bind
Common Ancestor	CA	A shared ancestor between 2 or more individuals
Expression phenotype		The expression patterns of genes **not** included in the fitness function (genes $0-11$)
Fitness phenotype		The expression patterns of genes included in the fitness function (genes $12, 13$)
Gene Regulatory Network	GRN	
Conserved interaction		Regulatory interaction which was present continuously in an ancestry trace for at least 5000 generations
Pruned network		A minimal GRN which is obtained by iteratively removing genomic elements from a genome and accepting the removal if fitness is not impacted
Ancestry trace		A trace back through evolutionary time from an individual to it's first ancestor (Fig. 1)
WUSCHEL	WUS	MADS box TF associated with the stem cell pool in the OC
CLAVATA3	CLV3	CLE TF associated with stem cell differentiation and proliferation in the CZ
Conserved non-coding sequence	CNS	A non-coding sequence which is found to be conserved within a group of orthologs
CNS landscape		The set of CNSs associated with a specific gene
genotype-phenotype map	GPM	The function mapping from genotype space to phenotype space

Table S2. Terms and abbreviations used in this work.
