# Supplementary material for: System drift in the evolution of plant meristem development
Source: PLoS Genet. 2026 Apr 3;22(4):e1012089. doi: 10.1371/journal.pgen.1012089 (PMC13075796; doi:10.1371/journal.pgen.1012089)
Supplement: Attachment — Submitted filename: 159775_1_rebuttal_3562941_t4xzrr.pdf [file pgen.1012089.s020.pdf]

Revision Plan

Manuscript number: RC-2025-03117
Corresponding author(s): Pjotr L. van der Jagt

1. General Statements

We would like to thank the referees for their time and effort in giving feedback on our work, and their overall positive attitude towards the manuscript. Most of the referees' points were of clarifying and textual nature. We have identified three points which we think require more attention in the form of additional analyses, simulations or significant textual changes:

1) Within the manuscript we state that conserved non coding sequences (CNSs) are a proxy for cis regulatory elements (CREs). We proceed to use these terms interchangeably without explaining the underlying assumption, which is inaccurate. To improve on this point we ensured in the new text that we are explicit about when we mean CNS or CRE. Secondly, we added a section to the discussion (‘*Limitations of CNSs as CREs*’) dedicated to this topic.
2) During stabilising selection (maintaining the target phenotype) DSD can occur fully neutrally, or through the evolution of either mutational or developmental robustness. We describe the evolutionary trajectories of our simulations as neutral once fitness mostly plateaued; however, as reviewer 3 points out, small gains in median fitness still occur, indicating that either development becomes more robust to noisy gene expression and tissue variation, and/or the GRNs become more robust to mutations. To discern between fully neutral evolution where the fitness distribution of the population does not change, and the higher-order emergence of robustness, we performed additional analysis of the given results. Preliminary results showed that many (near-)neutral mutations affect the mutational robustness and developmental robustness, both positively and negatively. To investigate this further we will run an additional set of simulations without developmental stochasticity, which will take about a week. These simulations should allow us to more closely examine the role of stabilising selection (of developmental robustness) in DSD by removing the need to evolve developmental robustness. Additionally, we will set up simulations in which we changed the total number of genes, and the number of genes under selection to investigate how this modelling choice influences DSD.
3) In the section on rewiring (‘*Network redundancy creates space for rewiring*’) we will analyse the mechanism allowing for rewiring in more depth, especially in the light of gene duplications and redundancy. We will extend this section with an additional analysis aimed to highlight how and when rewiring is facilitated.

We will describe the planned and incorporated revisions in detail below; we believe these have led to a greatly improved manuscript.

Kind regards,
Pjotr van der Jagt, Steven Oud and Renske Vroomans

Revision Plan

2. Description of the planned revisions

**Referee cross commenting (Reviewer 4)**
Reviewer 3's concern about DSD resulting from stabilising selection for robustness is something I missed -- this is important and should be addressed.
We understand this concern, and agree that we should be more thorough in our analysis of DSD by assessing the higher-order effects of stabilising selection on mutational robustness and/or environmental (developmental) robustness (McColgan & DiFrisco 2024).<br><br>We will 1) extend our analysis of fitness under DSD by computing the mutational and developmental robustness (similar to Figure 2F) over time for a number of ancestral lineages. By comparing these two measures over evolutionary time we will gain a much more fine grained image of the evolutionary dynamics and should be able to find adaptive trends through gain of either type of robustness. Preliminary results suggest that during the plateaued fitness phase both mutational robustness and developmental robustness undergo weak gains and losses, likely due to the pleiotropic nature of our GPM. Collectively, these weak gains and losses result in the gain observed in Figure S3. So, rather than fully neutral we should discern (near-)neutral regimes in which clear adaptive steps are absent, but in which the sum of them is a net gain. These are interesting findings we initially missed, and give insights into how this high-dimensional fitness landscape is traversed, and will be included in a future revised version of the manuscript.<br><br>2) We will run extra simulations without stochasticity to investigate DSD in the absence of adaptation through developmental robustness, and include the comparison between these and our original simulations in a future revised version.<br><br>Finally 3) we will address stabilising selection more prominently in the introduction and discussion to accommodate these additional simulations.
Reviewer 3 suggests that the model construction may favor DSD because there are many genes (14) of which only two determine fitness. I agree that some discussion on this point is warranted, though I am not sure enough is known about "the possible difference in constraints between the model and real development" for such a discussion to be on firm biological footing. A genetic architecture commonly found in quantitative genetic studies is that a small number of genes have large effects on the phenotype/fitness, whereas a very large number of genes have effects that are individually small but collectively large (see, e.g. literature surrounding the "omnigenic model" of complex traits). Implementing such an architecture is probably beyond the scope of the study here. More generally, would be natural to assume that the larger the number of genes, and the smaller the number of fitness-determining genes, the more likely DSD / re-wiring is to occur. That being said, I think the authors' choice of a 14-gene network is biologically defensible. It could be argued that the restriction of many modeling studies to small networks (often including just 3 genes) on the ground of convenience artificially ensures that DSD will not occur in these networks.

The choice of 14 genes does indeed stem from a compromise between constraining the number of available genes, but at the same time allowing for sufficient degrees of freedom and redundancy. We have added a ‘modelling choices’ section in the discussion in which we address this point. Additionally, it is important to note that, while the fitness criterion only measures the pattern of 2 genes, throughout the evolutionary lineage additional genes become highly important for the fitness of an individual, because these genes evolved to help generate the target pattern (see for example Figure 4); the other genes indeed reflect reviewer 4’s point that most genes have a small effect. Crucially, we observe that even the genes and interactions that are important for fitness undergo DSD.
Nevertheless, we think it is interesting to investigate this point of the influence of this particular modelling choice on the potential for DSD, and have set up an extra set of simulations with fewer gene types, and one with additional fitness genes.
Furthermore, we discuss the choice of our network architecture more in depth in a discussion section on our modelling choices: ‘*Modelling assumptions and choices*’.

Reviewer 1

The observation of DSD in the computational models remains rather high-level in the sense that no motifs, mechanisms, subgraphs, mutations or specific dynamics are reported to be associated to it ---with the exception of gene expression domains overlapping. Perhaps the authors feel it is beyond this study, but a Results section with a more in-depth "mechanistic" analysis on what enables DSD would (a) make a better case for the extensive and expensive computational models and (b) would push this paper to a next level. As a starting point, it could be nice to check Ohno's intuition that gene duplications are a creative "force" in evolution. Are they drivers of DSD? Or are TFBS mutations responsible for the majority of cases?

We agree that some mechanistic analysis would strengthen the manuscript, and will therefore extend the section ‘Network redundancy creates space for rewiring’ to address how this redundancy is facilitated. For instance, in the rewiring examples given in Figure 4 we can highlight how this new interaction emerges, if this is through a gene mutation followed by rewiring and loss of a redundant gene, or if the gain, redundancy and loss are all on the level of TFBS mutations. Effectively we will investigate which route of the three in the following schematic is most prominent:

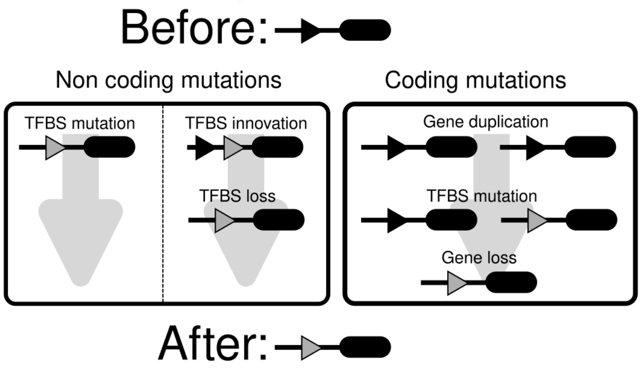

Additionally, we will do analysis on the different effects of the transcription dynamics for each of these routes. (note that this is not an exhaustive schematic, and combinations could be possible).
l171. You discuss an example here, would it be possible to generalize this analysis and quantify the amount of DSD amongst all cloned populations? And related question: of the many conserved interactions in Fig 4A, how many do the two clonal lineages share? None? All?
We agree that this is a good idea. In a new supplementary figure, we will show the number of times a conserved interaction gets lost, and a new interaction is gained as a metric for DSD in every cloned population.<br>The populations in Fig 4A are cloned at generation 50.000, any interaction starting before then and still present at a point in time is shared. Any interactions starting after 50.000 are unique (or independently gained at least).
- l269. What about phenotypic plasticity due to stochastic gene expression? Does it play a role in DSD in your model? I am thinking about https://pubmed.ncbi.nlm.nih.gov/24884746/ and https://pubmed.ncbi.nlm.nih.gov/21211007/
We agree that this is an interesting point which should be included into the discussion. Following the comments of reviewer 3 we have set up extra simulations to investigate this in more detail, we will make sure to include these citations in the revised discussion when we have the results of those simulations.
**Reviewer 3**
Issue One: Interpretation of fitness gains under stabilising selection<br><br>*A central issue concerns how the manuscript defines and interprets developmental systems drift (DSD) in relation to evolution on the fitness landscape. The authors define DSD as the conservation of a trait despite changes in its underlying genetic basis, which is consistent with the literature. However, the manuscript would benefit from clarifying the relationship between DSD, genotype-to-phenotype maps, and fitness landscapes. Very simply, we can say that (i) DSD can operate along neutral paths in the fitness landscape, (ii) DSD can operate along adaptive paths in the fitness landscape. During DSD, these neutral or adaptive paths along the fitness landscape are traversed by mutations that change the gene regulatory network (GRN) and consequent gene expression patterns whilst preserving the developmental outcome, i.e., the phenotype. While this connection between DSD and fitness landscapes is referenced in the introduction, it is not fully elaborated upon. A complete elaboration is critical because, when I read the manuscript, I got the impression that the manuscript claims that DSD is prevalent along neutral paths in the fitness landscape, not just adaptive ones. If I am wrong and this is not what the authors claim, it should be explicitly stated in the results and discussed. Nevertheless, claiming DSD operates along neutral paths is a much more interesting*

Review COMMONS

statement than claiming it operates along adaptive paths. However, it requires sufficient evidence, which I have an issue with.

The issue I have is about adaptations under stabilising selection. Stabilising selection occurs when there is selection to preserve the developmental outcome. Stabilising selection is essential to the results because evolutionary change in the GRN under stabilising selection should be due to DSD, not adaptations that change the developmental outcome. To ensure that the populations are under stabilising selection, the authors perform clonal experiments for 100,000 generations for 8 already evolved populations, 5 clones for each population. They remove 10 out of 40 clones because the fitness increase is too large, indicating that the developmental outcome changes over the 100,000 generations. However, the remaining 30 clonal experiments exhibit small but continual fitness increases over 100,000 generations. The authors claim that the remaining 30 are predominantly evolving due to drift, not adaptations (in the main text, line 137: "indicating predominantly neutral evolution", and section M: "too shallow for selection to outweigh drift"). The author's evidence for this claim is a mathematical analysis showing that the fitness gains are too small to be caused by beneficial adaptations, so evolution must be dominated by drift. I found this explanation strange, given that every clone unequivocally increases in fitness throughout the 100,000 generations, which suggests populations are adapting. Upon closer inspection of the mathematical analysis (section M), I believe it will miss many kinds of adaptations possible in their model, as I now describe.

The mathematical analysis treats fitness as a constant, but it's a random variable in the computational model. Fitness is a random variable because gene transcription and protein translation are stochastic (Wiener terms in Eqs. (1)-(5)) and cell positions change for each individual (Methods C). So, for a genotype G, the realised fitness F is picked from a distribution with mean μ_G and higher order moments (e.g., variance) that determine the shape of the distribution. I think these assumptions lead to two problems.

The first problem with the mathematical analysis is that F is replaced by an absolute number f_q, with beneficial mutations occurring in small increments denoted "a", representing an additive fitness advantage. The authors then take a time series of the median population fitness from their simulations and treat its slope as the individual's additive fitness advantage "a". The authors claim that drift dominates evolution because this slope is lower than a drift-selection barrier, which they derive from the mathematical analysis. This analysis ignores that the advantage "a" is a distribution, not a constant, which means that it does not pick up adaptations that change the shape of the distribution. Adaptations that change the shape of the distribution can be adaptations that increase robustness to stochasticity. Since there are multiple sources of noise in this model, I think it is highly likely that robustness to noise is selected for during these 100,000 generations.

The second problem is that the mathematical analysis ignores traits that have higher-order effects on fitness. A trait has higher-order effects when it increases the fitness of the lineage (e.g., offspring) but not the parent. One possible trait that can evolve in this model with higher-order effects is mutational robustness, i.e., traits that lower the expected mutational load of descendants. Since many kinds of mutations occur in this model (Table 2), mutational robustness may be also evolving.

Taken together, the analysis in Section M is set up to detect only immediate, deterministic additive gains in a single draw of fitness. It therefore cannot rule out weak but persistent adaptive evolution of robustness (to developmental noise and/or to mutations), and is thus insufficient evidence that DSD is occurring along neutral paths instead of adaptive paths. The small but monotonic fitness increases observed in all 40 clones are consistent with such adaptation (Fig. S3). The authors also acknowledge the evolution of robustness in lines 129-130 and 290-291, but the possibility of these adaptations driving DSD instead of neutral evolution is not discussed.

To address the issue I have with adaptations during stabilising selection, the authors should, at a minimum, state clearly in their results that DSD is driven by both the evolution of robustness and drift. Moreover, a paragraph in the discussion should be dedicated to why this is the case, and why it is challenging to separate DSD through neutral evolution vs DSD through adaptations such as those that increase robustness.

[OPTIONAL] A more thorough approach would be to make significant changes to the manuscript by giving sufficient evidence that the experimental clones are evolving by drift, or changing the model construction. One possible way to provide sufficient evidence is to improve the mathematical analysis. Another way is to show that the fitness distributions (both without and with mutations, like in Fig. 2F) do not significantly change throughout the 100,000 generations in experimental clones. It seems more likely that the model construction makes it difficult to separate the evolution of robustness from evolution by drift in the stabilising selection regime. Thus, I think the model should be constructed differently so that robustness against mutations and noise is much less likely to evolve after a "fitness plateau" is reached. This could be done by removing sources of noise from the model or reducing the kinds of possible mutations (related to issue two). In fact, I could not find justification in the manuscript for why these noise terms are included in the model, so I assume they are included for biological realism. If this is why noise is included, or if there is a separate reason why it is necessary, please write that in the model overview and/or the methods.

We agree that we should be more precise about whether DSD operates along neutral vs adaptive paths in the fitness landscape, and have expanded our explanation of this distinction in the introduction. We also agree that it is worthwhile to distinguish between neutral evolution that does not change the fitness distribution of the population (either through changes in developmental or mutational robustness), higher-order evolutionary processes that increase developmental robustness, and drift along a neutral path in the fitness landscape towards regions of greater connectivity, resulting in mutational robustness (as described in Huynen *et al.,* 1999). We have performed a preliminary analysis to identify changes in mutational robustness and developmental robustness over evolutionary time in the populations in which the maximum fitness has already plateaued. This analysis shows frequent weak gains and losses, in which clear adaptive steps are absent but a net gain can be seen in robustness, as consistent with higher-order fitness effects.

To investigate the role of stabilising selection more in depth we will run simulations without developmental noise in the form of gene expression noise and tissue connectivity variation, thus removing the effect of the evolution of developmental robustness. We will compare the

evolutionary dynamics of the GRNs with our original set of simulations, and include both these types of analyses in a supplementary figure of the revised manuscript.<br>Furthermore, we now discuss the limitations of the mathematical analysis with regard to adaptation vs neutrality in our simulations, in the supplementary section.
Issue two: The model construction may favour DSD<br><br>*In this manuscript, fitness is determined by the expression pattern of two types of genes (genes 12 and 13 in Table 1). There are 14 types of genes in total that can all undergo many kinds of mutations, including duplications (Table 2). Thus, gene regulatory networks (GRNs) encoded by genomes in this model tend to contain large numbers of interactions. The results show that most of these interactions have minimal effect on reaching the target pattern in high fitness individuals (e.g. Fig. 2F). A consequence of this is that only a minimal number of GRN interactions are conserved through evolution (e.g. Fig. 2D). From these model constructions and results from evolutionary simulations, we can deduce that there are very few constraints on the GRN. By having very few constraints on the GRN, I think it makes it easy for a new set of pattern-producing traits to evolve and subsequently for an old set of pattern-producing traits to be lost, i.e., DSD. Thus, I believe that the model construction may favour DSD.*<br>*I do not have an issue with the model favouring DSD because it reflects real multicellular GRNs, where it is thought that a minority fraction of interactions are critical for fitness and the majority are not. However, it is unknown whether the constraints GRNs face in the model are more or less constrained than real GRNs. Thus, it is not known whether the prevalence of DSD in this model applies generally to real development, where GRN constraints depend on so many factors. At a minimum, the possible difference in constraints between the model and real development should be discussed as a limitation of the model. A more thorough change to the manuscript would be to test the effect of changing the constraints on the GRN. I am sure there are many ways to devise such a test, but I will give my recommendation here.*<br>*[OPTIONAL] My recommendation is that the authors should run additional simulations with simplified mutational dynamics by constraining the model to N genes (no duplications and deletions), of which M out of these N genes contribute to fitness via the specific pattern (with M=2 in the current model). The authors should then test the effect of changing N and M independently, and how this affects the prevalence of DSD. If the prevalence of DSD is robust to changes in N and M, it supports the authors argument that DSD is highly prevalent in developmental evolution. If DSD prevalence is highly dependent on M and/or N, then the claims made in the manuscript about the prevalence of DSD must change accordingly. I acknowledge that these simulations may be computationally expensive, and I think it would be great if the authors knew (or devised) a more efficient way to test the effect of GRN constraints on DSD prevalence. Nevertheless, these additional simulations would make for a potentially very interesting manuscript.*
We agree that these modelling choices likely influence the potential for DSD. We think that our model setup, where most transcription factors are not under direct selection for a particular pattern, more accurately reflects biological development, where the outcome of the total

developmental process (a functional organism) is what is under selection, rather than each individual gene pattern. As also mentioned by the referee, in real multicellular development the majority of interactions is not crucial for fitness, similar to our model. We also observe that, as fitness increases, additional genes experience emergent selection for particular expression patterns or interaction structures in the GRN, resulting in their conservation. Nevertheless, we do agree that the effect of model construction on DSD is an unexplored avenue and this work lends itself to addressing this. We will run additional sets of simulations: one in which we reduce the size of the network (‘N’), and a second set where we double the number of fitness contributing genes (‘M’), and show the effect on the extent of DSD in a future supplementary figure.

3. Description of the revisions that have already been incorporated in the transferred manuscript

**Referee cross commenting (Reviewer 4)**
Overall I agree with the comments of Reviewer 1, 2 and 3. I note that reviewers 1, 3, and 4 each pointed out the difficulties with assuming that CNSs = CREs, so this needs to be addressed. Two reviewers (3 and 4) also point out problems with equating bulk RNAseq with a conserved phenotype.
We agree that caution is warranted with the assumption of CNSs = CREs. We have added a section to the discussion in which we discuss this more thoroughly, see ‘*Limitations of CNSs as CREs*’ in the revised manuscript.<br>Additionally, we made textual changes to the statement of significance, abstract and results to better reflect when we talk about CNSs or CREs.
I agree with Reviewer 1's hesitancy about the rhetorical framing of the paper potentially generalising too far from a computational model of plant meristem patterning.
We agree that the title should reflect the scope of the manuscript, and our short title reflects that better than ubiquitous, which implies we investigated beyond plant (meristem) development. We have changed the title in the revised version, to ‘*System drift in the evolution of plant meristem development*’.
**Reviewer 1**
It is system drift, not systems drift (see True and Haag 2001). No 's' after system.
Thank you for catching this – we corrected this throughout.
- I am afraid I have a problem with the manuscript title. I think "Ubiquitoes" is misplaced, because it strongly suggests you have a long list of case studies across plants and animals, and some quantification of DSD in these two kingdoms. That would have been an interesting result, but it is not what you report. I suggest something along the lines of "System drift in the

Review COMMONS

evolution of plant meristem development", similar to the short title used in the footer.<br>- Alternatively, the authors may aim to say that DSD happens all over the place in computational models of development? In that case the title should reflect that the claim refers to modeling. (But what then about the data analysis part?)
As remarked in the summary (point 2), we agree with this assessment and have changed the title to '*System drift in the evolution of plant meristem development*''
Multiple times in the Abstract and Introduction the authors make statements on "cis-regulatory elements" that are actually "conserved non-coding sequences" (CNS). Even if it is not uncommon for CNSs to harbor enhancers etc., I would be very hesitant to use the two as synonyms. As the authors state themselves, sequences, even non-coding, can be conserved for many reasons other than CREs. I would ask the authors to support better their use of "CREs" or adjust language. As roughly stated in their Discussion (lines 310-319), one way forward could be to show for a few CNS that are important in the analysis (of Fig 5), that they have experimentally-verified enhancers. Is that do-able or a bridge too far?
We changed the text such that we use CNS instead of CRE when discussing the bioinformatic analysis. Additionally we added a section in the discussion to clarify the relationship between CNS and CRE.
line 7. evo-devo is jargon
We changed this to '*…evolution of development (evo-devo) research…*'
l9. I would think "using a computational model and data analysis"
Yes, corrected.
l13. Strictly speaking you did not look at CREs, but at conserved non-coding sequences.
Indeed, we changed this to CNS.
l14. "widespread" is exaggerated here, since you show for a single organ in a handful of plant species. You may extrapolate and argue that you do not see why it should not be widespread, but you did not show it. Or tie in all the known cases that can be found in literature..
We understand that 'widespread' seems to suggest that we have investigated a broader range of species and organs. To be more accurate we changed the wording to 'prevalent'.

Revision Plan

l16. "simpler" than what?
We added the example of RNA folding.
l27. Again the tension between CREs and non-coding sequence.
Changed to conserved non coding sequence.
l28. I don't understand the use of "necessarily" here.
This is indeed confusing and unnecessary, removed
l34-35. A very general biology statement is backed up by two modeling studies. I would have expected also a few based on comparative analyses (e.g., fossils, transcriptomics, etc).
We added extra citations and a discussion of more experimental work
l36. I was missing the work on "phenogenetic drift" by Weiss; and Pavlicev & Wagner 2012 on compensatory mutations.
Changed the text to:<br>*This phenomenon is called developmental system drift (DSD) (True and Haag, 2001; McColgan and DiFrisco, 2024), or phenogenetic drift (Weiss and Fullerton, 2000), and can occur when multiple genotypes which are separated by few mutational steps encode the same phenotype, forming a neutral (Wagner, 2008a; Crombach et al., 2016); or adaptive path (Johnson and Porter, 2007; Pavlicev and Wagner, 2012) .*
l38. Kimura and Wagner never had a developmental process in mind, which is much bigger than a single nucleotide or a single gene, respectively. First paper that I am aware of that explicitly connects DSD to evolution on genotype networks is my own work (Crombach 2016), since the editor of that article (True, of True and Haag 2001) highlighted that point in our communications.
Added citation and moved Kimura to the theoretical examples of protein folding DSD.
l40. While Hunynen and Hogeweg definitely studied the GP map in many of their works, the term goes back to Pere Alberch (1991).
Added citation.
l54-55. I'm missing some motivation here. If one wants to look at multicellular structures that display DSD, vulva development in C. elegans and related worms is an "old" and extremely well-studied example. Also, studies on early fly development by Yogi Jaeger and his co-workers are not multicellular, but at least multi-nuclear.

Obviously these are animal-based results, so to me it would make sense to make a contrast animal-plant regarding DSD research and take it from there.
Indeed, DSD has been found in these species and we now reference some of this work; the principle is better known in animals. Nevertheless, within the theoretical literature there is a continuing debate on the importance/extent of DSD.<br>Changed text:<br>*'For other GPMs, such as those resulting from multicellular development, it has been suggested that complex phenotypes are sparsely distributed in genotype space, and have low potential for DSD because the number of neutral mutations anti-correlates with phenotypic complexity (Orr, 2000; Hagolani et al., 2021). On the other hand, theoretical and experimental studies in nematodes and fruit flies have shown that DSD is present in a phenotypically complex context (Verster et al., 2014; Crombach et al., 2016; Jaeger, 2018). It therefore remains debated how much DSD actually occurs in species undergoing multicellular development. DSD in plants has received little attention. One multicellular structure which …'*
l66-86. It is a bit of a style-choice, but this is a looong summary of what is to come. I would not have done that. Instead, in the Introduction I would have expected a bit more digging into the concept of DSD, mention some of the old animal cases, perhaps summarize where in plants it should be expected. More context, basically.
We extended the paragraph on empirical examples of DSD by adding the animal cases and condensed our summary.
l108. Could you quantify the conserved interactions shared between the populations? Or is each simulation so different that they are pretty much unique?
Each simulation here is independent of the other simulations, so a per interaction comparison would be uninformative. After cloning they do share ancestry, but that is much later in the manuscript and here the quantification of the conserved interactions would be the inverse of the divergence as shown in, for instance Figure 3B.
l169. "DSD driving functional divergence" needs some context, since DSD is supposed to not affect function (of the final phenotype). Or am I misunderstanding?
This is indeed a confusing sentence. We mean to say that DSD allows for divergence to such an extent that the underlying functional pathway is changed. So instead of a mere substitution of the underlying network, in which the topology and relative functions stay conserved, a different network structure is found. We have modified the line to read "*Taken together, we found that DSD can drive functional divergence in the underlying GRN resulting in novel spatial expression dynamics of the genes not directly under selection.*"
l176. Say which interaction it is. Is it 0->8, as mentioned in the next paragraph?

It is indeed 0->8, we have clarified this in the text.
l197. Bulk RNAseq has the problem of averaging gene expression over the population of cells. How do you think that impacts your test for rewiring? If you would do a similar "bulk RNA" style test on your computational models, would you pick up DSD?
The rewiring is based on the CNSs, whereas the RNAseq is used as phenotype, so it does not impact the test for rewiring.<br>The averaging of bulk RNAseq does however, mean that we cannot show conservation/divergence of the phenotype within the tissues, only between the different tissues.<br>The most important implication of doing this in our model would be the definition of the 'phenotype' which undergoes DSD. Currently the phenotype is a gene expression pattern on a cellular level, for bulk RNA this phenotype would change to tissue-level gene expression. This change in what we measure as phenotype implicates how we interpret our results, but would not hinder us in picking up DSD, it just has a different meaning than DSD on a cellular - and single tissue scale.<br>We added clarification of the roles of the datasets at the start of the paragraph.<br>*'The Conservatory Project collects conserved non-coding sequences (CNSs) across plant genomes, which we used to investigate the extent of GRN rewiring in flowering plants. Schuster et al. measured gene expression in different homologous tissues of several species via bulk RNAseq, which we used to test for gene expression (phenotype) conservation, and how this relates to the GRN rewiring inferred from the CNSs.'*
l202. I do not understand the "within" of a non-coding sequence within an orthogroup. How are non-coding sequences inside an orthogroup of genes?
We clarify this sentence by saying '*A CNS is defined as a non-coding sequence conserved within the upstream/downstream region of genes within an orthogroup*', to more clearly separate the CNS from the orthogroup of genes. We also updated Figure 5A to reflect this better.
l207-217. This paragraph is difficult to read and would benefit of a rephrasing. Plant-specific jargon, numbers do not add up (line 211), statements are rather implicit (9 deeply conserved CNS are the 3+6? Where do I see them in Fig 5B? And where do I see the lineage-specific losses?).
We added extra annotations to the figure to make the plant jargon (angiosperm, eudicot, Brassicaceae) clear, and show the loss more clearly in the figure. We also clarified the text by splitting up 9 to 3 and 6.
l223. Looking at the shared CNS between SEP1-2, can you find a TF binding site or another property that can be interpreted as regulatory importance?

Reliably showing an active TF binding site would require experimental data, which we don't have. We do mention in the discussion the need for datasets which could help address this gap.
l225. My intuition says that the continuity of the phenotype may not be necessary if its loss can be compensated for somehow by another part of the organism. I.e., DSD within DSD. It is a poorly elaborated thought, I leave it here for your information. Perhaps a Discussion point?
Although very interesting we think this discussion might be outside of the scope of this work, and would benefit from a standalone discussion – especially since the capacity for such compensation might differ between animals and plants (which are more "modular" organisms). This is our interpretation:<br>First, let's take a step back from 'genotype' and 'phenotype' and redefine DSD more generally: in a system with multiple organisational levels, where a hierarchical mapping between them exists, DSD is changes on one organisational level which do not alter the outcome of the 'higher' organisational level. In other words, DSD can exist any many-to-one mapping in which a set of many (which map to the same one) are within a certain distance in space, which we generally define as a single mutational step.<br>Within this (slightly) more general definition we can extend the definition of DSD to the level of phenotype and function, in which phenotype describes the 'many' layer, and multiple phenotypes can fulfill the same function. When we are freed from the constraint of 'genotype' and 'phenotype', and DSD is defined at the level of this mapping, than it becomes an easy exercise to have multiple mappings (genotype→phenotype→function) and thus 'DSD within DSD'.
l233. "rarely"? I don't see any high Pearson distances.
True in the given example there are no high Pearson distances, however some of the supplementary figures do so rarely felt like the most honest description. We changed the text to refer to these supplementary figures.
Fig 4. Re-order of panels? I was expecting B at C and vice versa.
Agreed, we swapped the order of the panels
Fig 5B. Red boxes not explained. Mention that it is an UpSetplot?
We added clarification to the figure caption.
Fig 5D. It would be nice to quantify the minor and major diffs between orthologs and paralogs.
We quantify the similarities (and thus differences) in Figure F, but we do indeed not show orthologs vs paralogs explicitly. We have extended Figure F to distinguish which comparisons are between orthologs vs paralogs with different tick marks, which shows their different distributions quite clearly.

- l247. Over-generalization. In a specific organ of plants...
Changed to vascular plant meristem.
- l249. Where exactly is this link between diverse expression patterns and the Schuster dataset made? I suggest the authors to make it more explicit in the Results.
We are slightly overambitious in this sentence. The Schuster dataset confirms the preservation of expression where the CNS dataset shows rewiring. That this facilitates diversification of expression patterns in traits not under selection is solely an outcome of the computational model. We have changed the text to reflect this more clearly.
- l268. Final sentence of the paragraph left me puzzled. Why talk about opposite function?
The goal here was to highlight regulatory rewiring which, in the most extreme case, would achieve an opposite function for a given TF within development. We agree that this was formulated vaguely so we rewrote this to be more to the point.<br>*These examples demonstrate that whilst the function of pathways is conserved, their regulatory wiring often is not.*
- l269. What about time scales generated by the system? Looking at Fig 2C and 2D, the elbow pattern is pretty obvious. That means interactions sort themselves into either short-lived or long-lived. Worth mentioning?
Added a sentence to highlight this.
*- l291. Evolution in a *constant* fitness landscape increases robustness.*
Changed
- l296. My thoughts, for your info: I suspect morphogenesis as single parameters instead of as mechanisms makes for a brittle landscape, resulting in isolated parts of the same phenotype.
We agree, and now include citations to different models in which morphogenesis evolves which seem to display a more connected landscape.
**Reviewer 2**
Every computational model necessarily makes some simplifying assumptions. It would be nice if the authors could summarise in a paragraph in the Discussion the main assumptions made by their model, and which of those are most worth revisiting in future studies. In the current draft, some assumptions are described in different places in the manuscript, which makes it hard for a non-expert to evaluate the limitations of this model.
We added a section to the discussion: *'Modelling assumptions and choices'*

I did not find any mention of potential energetic constraints or limitations in this model. For example, I would expect high levels of gene expression to incur significant energy costs, resulting in evolutionary trade-offs. Could the authors comment on how taking energy limitations into account might influence their results?
This would put additional constraints on the evolution/fitness landscape. Some paths/regions of the fitness landscape which are currently accessible will not be traversable anymore. On the other hand, an energy constraint might reduce certain high fitness areas to a more even plane and thus make it more traversable. During analysis of our data there were no signs of extremely high gene expression levels.
Figure 3C lists Gene IDs 1, 2, 8, and 11, but the caption refers to genes 1, 2, 4, and 11.
Thank you for catching this.
**Reviewer 3**
1. The authors present an analysis correlating conserved non-coding sequence (CNS) composition with gene expression to investigate developmental systems drift. One flaw of this analysis is that it uses deeply conserved sequences as a proxy for the entire cis-regulatory landscape. The authors acknowledge this flaw in the discussion.<br>*Another potential flaw is equating the bulk RNA-seq data with a conserved phenotype. In lines 226-227 of the manuscript, it is written that "In line with our computational model, we compared gene expression patterns to measure changes in phenotype." I am not sure if there is an equivalence between the two. In the computational model, the developmental outcome determining fitness is a spatial pattern, i.e., an emergent product of gene expression and cell interactions. In contrast, the RNA-seq data shows bulk measurements in gene expression for different organs. It is conceivable that, despite having very similar bulk measurements, the developmental outcome in response to gene expression (such as a spatial pattern or morphological shape) changes across species. I think this difference should be explicitly addressed in the discussion. The authors may have intended to discuss this in lines 320-326, although it is unclear to me.*
It is correct that the CNS data and RNA-seq data has certain limitations, and the brief discussion of some of these limitations in lines 320-326 is not sufficient. We have been more explicit on this point in the discussion.<br>*The gene expression data used in this study represents bulk expression at the organ level, such as the vegetative meristem (Schuster et al., 2024). This limits our analysis of the phenotypic effects of rewiring to comparisons between organs, which is different to our computational simulations where we look at within organ gene expression. Additionally, the bulk RNA-seq does not allow us to discern whether the developmental outcome of similar gene expression is the same in all these species. More fine-grained approaches, such as single-cell RNA sequencing or spatial transcriptomics, will provide a more detailed understanding of how gene expression is modulated spatially and temporally within complex tissues of different organisms, allowing for a closer alignment between computational predictions and experimental observations.*

2. Can the authors justify using these six species in the discussion or the results? Are there any limitations with choosing four closely related and two distantly related species for this analysis, in contrast to, say, six distantly related species? If so, please elaborate in the discussion.

The use of these six species is mainly limited by the datasets we have available. Nevertheless, the combination of four closely related species, and two more distantly related species gives a better insight into the short vs long term divergence dynamics than six distantly related species would. We have noted this when introducing the datasets:
This set of species contains both closely (A. thaliana, A. lyrata, C. rubella, E. salsugineum) and more distantly related species (M. truncatula, B. distachyon), which should give insight in short and long term divergence.

3. In Figure S7, some profiles show no conservation across the six species. Can we be sure that a stabilising selection pressure conserves any CNSs? Is it possible that the deeply conserved CNSs mentioned in the main text are conserved by chance, given the large number of total CNSs? A brief comment on these points in the results or discussion would be helpful.

In our simulations, we find that even CREs that were under selection for a long time can disappear; however, in our neutral simulations, CREs were not conserved, suggesting that deep conservation is the result of selection. When it comes to CNSs, the assumption is that they often contain CREs that are under selection.We have added a more elaborate section on CNSs in the discussion. See '*Limitations of CNSs as CREs*'

4. Line 7-8: I thought this was a bit difficult to read. The connection between (i) evolvability of complex phenotypes, (ii) neutral/beneficial change hindered by deleterious mutations, and (iii) DSD might not be so simple for many readers, so I think it should be rewritten. The abstract was well written, though.

We made the connection to DSD and evolvability clearer and removed the specific mutational outcomes:
A key open question in evolution of development (evo-devo) is the evolvability of complex phenotypes. Developmental system drift (DSD) may contribute to evolvability by exploring different genotypes with similar phenotypic outcome, but with mutational neighbourhoods that have different, potentially adaptive, phenotypes. We investigated the potential for DSD in plant development using a computational model and data analysis.

5. Line 274 vs 276: Is there a difference between regulatory dynamics and regulatory mechanisms?

No, we should use the same terminology. We have changed this to be clearer.

6. Figure S4: Do you expect the green/blue lines to approach the orange line in the long term? In some clonal experiments, it seems like it will. In others, it seems like it has plateaued. Under continual DSD, I assume they should converge. It would be interesting to see simulations run sufficiently long to see if this occurs.

In principle yes, however this might take a considerable amount of time given that some conserved interactions take >75000 generations to be rewired.
7. Line 27: Evolutionarily instead of evolutionary?
Changed
8. Line 67-68: References in brackets?
Changed
9. Line 144: Capitalise "fig"
Changed
10. Fig. 3C caption: correct "1, 2, 4, 11" (should be 8)
Changed
11. Line 192: Reference repeated
Changed
12. Fig. 5 caption: Capitalise "Supplementary figure"
Changed
13. Line 277: Correct "A previous model Johnson.."
Changed
14. Line 290: Brackets around reference
Changed
15. Line 299: Correct "will be therefore be"
Changed
16. Line 394: Capitalise "table"
Changed
17. Line 449: Correct "was build using"
Changed
18. Fig. 5B: explain the red dashed boxes in the caption

Added explanation to the caption
19. Some of the Figure panels might benefit from further elaboration in their respective captions, such as 3C and 5F.
Improved the figure captions.
**Reviewer 4**
Statement of significance. The logical connection between the first two sentences is not clear. What does developmental system drift have to do with neutral/beneficial mutations?
This is indeed an unclear jump. Changed such that the connection between evolvability of complex phenotypes and DSD is more clear:<br>*A key open question in evolution of development (evo-devo) is the evolvability of complex phenotypes. Developmental system drift (DSD) contributes to evolvability by exploring different genotypes with similar phenotypic outcome, but with mutational neighbourhoods that have different, potentially adaptive, phenotypes..We investigated the potential for DSD in plant development using a computational model and data analysis.*
l 41 - "DSD is found to ... explain the developmental hourglass." Caution is warranted here. Wotton et al 2015 claim that "quantitative system drift" explains the hourglass pattern, but it would be more accurate to say that shifting expression domains and strengths allows compensatory regulatory change to occur with the same set of genes (gap genes). It is far from clear how DSD could explain the developmental hourglass pattern. What does DSD imply about the causes of differential conservation of different developmental stages? It's not clear there is any connection here.
We should indeed be more cautious here. DSD is indeed not in itself an explanation of the hourglass model, but only a mechanism by which the developmental divergence observed in the hourglass model could have emerged. As per Pavlicev and Wagner, 2012, compensatory changes resulting from other shifts would fall under DSD, and can explain how the patterning outcome of the gap gene network is conserved. However, this does not explain why some stages are under stronger selection than others. We changed the text to reflect this.<br>‘*...be a possible evolutionary mechanism involved in the developmental hourglass model (Wotton et al., 2015; Crombach et al., 2016)...*’
ll 51-53 - "Others have found that increased complexity introduces more degrees of freedom, allowing for a greater number of genotypes to produce the same phenotype and potentially allowing for more DSD (Schiffman and Ralph, 2022; Greenbury et al., 2022)." Does this refer to increased genomic complexity or increased phenotypic complexity? It is not clear that increased phenotypic complexity allows a greater number of genotypes to produce the same phenotype. Please explain further.

The paragraph discusses complexity in the GPM as a whole, where the first few examples in the paragraph regard phenotypic complexity, and the ones in l51-53 refer to genomic complexity. This is currently not clear so we clarified the text.<br>*'For other GPMs, such as those resulting from multicellular development, it has been suggested that complex phenotypes are sparsely distributed in genotype space, and have low potential for DSD because the number of neutral mutations anti-correlates with phenotypic complexity (Orr, 2000; Hagolani et al., 2021). Others have found that increased genomic complexity introduces more degrees of freedom, allowing for a greater number of genotypes to produce the same phenotype and potentially allowing for more DSD (Schiffman and Ralph, 2022; Greenbury et al., 2022).'*
It was not clear why some gene products in the model have the ability to form dimers. What does this contribute to the simulation results? This feature is introduced early on, but is not revisited. Is it necessary?<br>*Fitness. The way in which fitness is determined in the model was not completely clear to me.*
Dimers are not necessary, but as they have been found to play a role in actual SAM development we added them to increase the realism of the developmental simulations. In some simulations the patterning mechanism involves the dimer, in others it does not, suggesting that dimerization is not essential for DSD.<br>We have made changes to the methods to clarify fitness.
Lines 103-104 say: "Each individual is assigned a fitness score based on the protein concentration of two target genes in specific regions of the SAM: one in the central zone (CZ), and one in the organizing center (OC)." How are these regions positionally defined in the simulation?
We have defined bounding boxes to define cells as either CZ, OC or both. We have added these bounds in the figure description and more clearly in the revised methods.
F, one reads (l. 385): "Fitness depends on the correct protein concentration of the two fitness genes in each cell, pcz and poc respectively." This sounds like fitness is determined by the state of all cells rather than the state of the two specific regions of the SAM. Please clarify.
A fitness penalty is given for incorrect expression so it is true that the fitness is determined by the state of all cells. We agree that it is phrased unclearly and have clarified this in the text.
The authors use conserved non-coding sequences as a proxy for cis-regulatory elements. More specification of how CNSs were assigned to an orthogroup seems necessary in this section. Is assignment based on proximity to the coding region? Of course the authors will appreciate that regulatory elements can be located far from the gene they regulate. This data showed extensive gains and losses of CNS. It might be interesting to consider how much of this is down to transposons, in which case rapid rearrangement is not unexpected. A potential problem with the claim that the data supports the simulation results follows from the fact that DSD is genetic divergence despite trait conservation, but conserved traits appear to have only been defined or identified in the case of the SEP genes. It can't be ruled out that divergence in

CNSs and in gene expression captured by the datasets is driven by straightforward phenotypic adaptation, thus not by DSD. Further caution on this point is needed.
CNSs are indeed assigned based on proximity up to 50kb, the full methods are described in detail in Hendelman et al., (2021). CREs can be located further than 50kb, but evidence suggests that this is rare for species with smaller genomes.<br>In the cases where both gene expression and the CNSs diverged it can indeed not be ruled out that there has been phenotypic adaptation. We clarified in the text that the lower Pearson distances are informative for DSD as they highlight conserved phenotypes.
l. 290-291 - "However, evolution has been shown to increase mutational robustness over time, resulting in the possibility for more neutral change." It is doubtful that there is any such unrestricted trend. If mutational robustness only tended to increase, new mutations would not affect the phenotype, and phenotypes would be unable to adapt to novel environments. Consider rethinking this statement.
We have reformulated this statement, since it is indeed not expected that this trend is indefinite. Infinite robustness would indeed result in the absence of evolvability; however, it has been shown for other genotype-phenotype maps that mutational robustness, where a proportion of mutations is neutral, aids the evolution of novel traits. The evolution of mutational robustness also depends on population size and mutation rate. This trend will, most probably, also be stronger in modelling work where the fitness function is fixed, compared to a real life scenario where 'fitness' is much less defined and subject to continuous change. We added '*constant*' to the fitness landscape to highlight this disparity.
ll. 316-317 "experimental work investigating the developmental role of CREs has shown extensive epistasis - where the effect of a mutation depends on the genetic background - supporting DSD." How does extensive epistasis support DSD? One can just as easily imagine scenarios where high interdependence between genes would prevent DSD from occurring. Please explain further.
We should be more clear. Experimental work has shown that the effect of mutating a particular CRE strongly depends on the genetic background, also known as epistasis. Counterintuitively, this indirectly supports the presence of DSD, since it means that different species or strains have slightly different developmental mechanisms, resulting in these different mutational effects. We have shown how epistatic effects shift over evolutionary time.
Overall I found the explanation of the Methods, especially the formal aspects, to be unclear at times and would recommend that the authors go back over the text to improve its clarity.
We rewrote parts of the methods and some of the equations to be more clear and cohesive throughout the text.
C. Tissue Generation. Following on the comment on fitness above, it would be advisable to provide further details on how cell positions are defined. How much do the cells move over the

Review COMMONS

course of the simulation? What is the advantage of modelling the cells as "springs" rather than as a simple grid?
The tissue generation is purely a process to generate a database of tissue templates: the random positions, springs and voronoi method serve the purpose of having similar but different tissues to prevent unrealistic overfitting of our GRNs on a single topology. For each individual's development however, only one, unchanging template is used. We clarified this in the methods.
E. Development of genotype into phenotype. The diffusion term in the SDE equations is hard to understand as no variable for spatial position (x) is included in the equation. It seems this equation should rather be an SPDE with a position variable and a specified boundary condition (i.e. the parabola shape). In eq. 5 it should be noted that the Wi are independent. Also please justify the choice of how much noise/variance is being stipulated here.
We have rewritten parts of this section for clarity and added citations.
F. Fitness function. I must say I found formula 7 to be unclear. It looks like fi is the fitness of cell(s) but, from Section G, fitness is a property of the individual. It seems formula 7 should define fi as a sum over the cell types or should capture the fitness contribution of the cell types.
Correct. We have rewritten this equation. We'll define fi as the fitness contribution of a cell, F as the sum of fi, so the fitness of an individual, and use F in function 8.
What is the basis for the middle terms (fractions) in the equation? After plugging in the values for pcz and poc, this yields a number, but how does that number assign a cell to one of the types? If a reviewer closely scrutinizing this section cannot make sense of it, neither will readers. Please explain further.
The cell type is assigned based on the spatial location of the cell, and the correct fitness function for each of these cell types is described in this equation. We have clarified the text and functions.
A minor note: it would be best practice not to re-use variables to refer to different things within the same paper. For example p refers to protein concentration but also probability of mutation.
Corrected

4. Description of analyses that authors prefer not to carry out
